# Health policy dialogue: experiences from Africa

**DOI:** 10.1186/s12913-016-1447-x

**Published:** 2016-07-18

**Authors:** Delanyo Dovlo, Martin Ekeke Monono, Tarcise Elongo, Juliet Nabyonga-Orem

**Affiliations:** Health Systems and Services Cluster, World Health Organization Regional Office for Africa, B.P. 06 Brazzaville, Congo

## Background

The high volume of investments in health seen in Africa over the last decade, mainly through global health initiatives [1], has enabled significant improvements in some health indicators in many of the countries [2]. However, these gains have not always translated into sustained improvements in the health sector’s capacity or health system’s performance [3]. Countries in the World Health Organization African Region continue to face serious challenges among which are major inadequacies in the health workforce, low levels of health spending with significant out of pocket payments, weak procurement and supply systems, poor information and monitoring systems, [4] and weak community engagement [5]. The 2014–2016 Ebola virus disease outbreak in West Africa illustrated the importance of a functional health system in sustaining health service delivery and economic and social development amidst a crisis [6, 7]. Weaknesses in health sectors’ organisation and governance continue to undermine the efficiency of health service delivery [8].

The adoption of the 17 Sustainable Development Goals in September 2015 raises questions about the countries’ capacity to attain these new and broader goals when most of them failed to reach the targets of the Millennium Development Goals [4]. Realising the Sustainable Development Goals requires making good policy and strategic choices in the effort to achieve universal health coverage. Effective country stewardship, and governance and leadership of the health sector, along with ownership of the goals, will be needed to mobilise relevant stakeholders and to guide domestic and external investments towards the agreed goals. This will also require strengthening of partnerships, effective intersectoral engagement and collaboration, and community engagement.

An important element in moving forward will be government leadership and coordination of effective policy dialogues and strategic planning to arrive at well-considered and evidence-based national health policies and sector strategic plans that are owned and supported by all stakeholders. In Africa, with its looming non-communicable disease catastrophe and vibrant social, demographic and economic growth, sophisticated policy dialogue and evidence-based choices will increasingly be essential. The challenge of getting from research evidence to policy decisions will fare better with effective policy dialogue.

The papers in this supplement review present recent experiences from some countries in the World Health Organization Africa Region from a programme to expand and support policy dialogue in the health sector. The experience has been illuminating with regard to the factors relating to the political players and processes, and the subjective elements that fuel policy debates. The themes of the papers were generated based on the critical questions on how policy dialogue would work in the various contexts found in a set of low income countries that receive substantial external aid inputs and therefore see significant influence in policy development and decision-making processes.

It was important to determine what the enablers of effective policy dialogue were in those contexts and what incentives or motivators guided the stakeholders towards a viable policy dialogue. The papers highlight the fact that policy dialogue requires resources in terms of time, space, evidence, good facilitation, dialogue skills and advocacy. Informal and formal platforms need to be employed for consensus building, and potential influence and power imbalance need to be anticipated and mitigated. Often, as with any policy change or choice, there will be winners and losers—both metaphorically and concretely—in terms of access to health services and shifts in other interests. Understanding the impact of choices made should be a critical part of the dialogue processes.

Timely and innovative dissemination of relevant information is needed to ensure that the dialogue is informed. Generally, capacity, knowledge and information are unevenly distributed among the stakeholders, including (and in particular) the beneficiaries of the policy results and changes. That is one area where initial assessments are needed to ensure that engagement in the dialogues and other policy processes is well informed, and not just by the presence of relevant actors but also by effective interaction to enable the voice of the marginalised to be heard. In situations of funding through general budget support, external catalytic funds can help to sustain policy dialogue processes and implementation of agreed critical follow-up actions. These are some of the lessons from the process that the World Health Organization Regional Office for Africa undertook with some of its Member States.

Policy dialogue in the era of disease-focused global health initiatives faces challenges and questions: what concrete results are expected from a policy dialogue process? how can we tell how many children’s lives were saved? and how can we directly link this political and often qualitative process to concrete health results and benefits? At the very least, it will be necessary to determine the key processes and intermediary results that can be recognised as salutary to the effective governance of health systems. Other challenges are on (1) how to sustain the use of the tools, processes and mechanisms that result in good policy dialogues and decisions, (2) how we can build and sustain effective community engagement so as to embed policy dialogues in societal processes, and (3) how policy dialogues can become institutionalised in sector mechanisms and review processes. These questions point to the areas for further research.

The World Health Organization in the Africa Region values the extensive learning for its staff, ministry of health officials, development partners and other stakeholders from the experience the policy dialogue programme has generated. These lessons will be best sustained by being translated into guidance for countries on the development of health policy and strategic plans and designing of stakeholders’ engagement mechanisms. Training in facilitating health policy dialogues should be a critical component of any health policy course and any senior public health qualification.

While it is a difficult process fraught with many competing interests and challenges, effective policy dialogue is essential to create the policy context needed in Africa to achieve the Sustainable Development Goals.
